# Detection of *Cucumber green mottle mosaic virus* in low-concentration virus-infected seeds by improved one-step pre-amplification RT-qPCR

**DOI:** 10.1186/s13007-022-00901-2

**Published:** 2022-05-26

**Authors:** Yin Xinying, Li Xin, Yang Lili, Zheng Qiuyue, Piao Yongzhe, Cao Jijuan

**Affiliations:** 1 Key Laboratory of Biotechnology and Bioresources Utilization of Ministry of Education, Dalian Minzu University, Dalian, 116600 China; 2 Dalian Customs Technology Center, Dalian, 116001 China

**Keywords:** Low concentration virus-infected seed, *Cucumber green mottle mosaic virus* (CGMMV), One-step pre-amplification RT-qPCR, Sensitivity, Detection

## Abstract

**Background:**

Seeds were an important medium for long-distance transmission of plant viruses. Therefore, appropriate, more sensitive methods for detecting low concentrations of virus-infected in seeds were crucial to ensure the quality of seed lots. In this study, we have developed a one-step pre-amplification reverse transcription quantitative PCR (RT-qPCR) assay based on the TaqMan technology to detect *Cucumber green mottle mosaic virus* (CGMMV) in zucchini seeds.

**Result:**

Seed powder samples with simulated CGMMV-infected at a low concentration were prepared (the mass ratio 1:900 and 1:1000), and their uniformity were verified using one-step pre-amplification RT-qPCR. We used one-step pre-amplification RT-qPCR to detect CGMMV in low-concentration virus-infected seeds and compared this method with universal RT-qPCR and double antibody sandwich–enzyme-linked immunosorbent (DAS–ELISA) assay, the main methods used for virus detection in seeds. The minimum limit of detection (LOD) of the improved one-step pre-amplification RT-qPCR assays for simulated CGMMV-infected seeds in large lots seeds samples were 0.1%.

**Conclusions:**

One-step pre-amplification RT-qPCR assays could reliably and stably detected a single CGMMV-infected seed in 1000 seeds and demonstrated a higher detection sensitivity than universal RT-qPCR (infected seeds versus healthy seeds 1:900) and DAS–ELISA assay (infected seeds versus healthy seeds 1:500). Our improved one-step pre-amplification RT-qPCR assay have proved to be very suitable for the analysis of large seed lots.

**Graphical Abstract:**

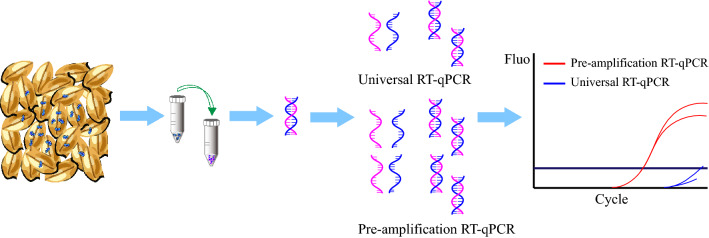

**Supplementary Information:**

The online version contains supplementary material available at 10.1186/s13007-022-00901-2.

## Background

Seeds can be important vehicles for long-distance transmission of plant viruses and can be used as a bridge for viruses to survive between growing seasons [1–3]. Therefore, adequate analytical methods are crucial for ensuring that seed lots are free of viruses [4]. The double antibody sandwich–enzyme-linked immunosorbent assay (DAS–ELISA) have been the main method for detecting viruses in seeds and were recognized by the International Seed Health Initiative and the International Seed Testing Association (ISTA) [5]. DAS–ELISA can be used to analyze a large number of samples, but it has poor detection sensitivity. In addition, determination of biological characteristics or germination test are also commonly used in seed analysis [6]; however, these methods are labor intensive and need specialized facilities [7]. Therefore, it is necessary to continuously develop and improve virus detection methods in seeds, especially highly sensitive detection methods, as the concentration of viruses in seeds is usually lower than that in other plant tissues [8,9].

Compared with other molecular techniques, reverse transcription quantitative PCR (RT-qPCR) based on the TaqMan technology has higher sensitivity and specificity [10]. It has been widely used in the detection of plant viruses such as *Squash leaf curl virus* (SLCV) [11], *Papaya ringspot virus* (PRSV) [12], *Papaya leaf distortion mosaic virus* (PLDMV) [12], *Cucurbit chlorotic yellows virus* (CCYV) [13], *Cucurbit yellow stunting disorder virus* (CYSDV) [13], and *Cucumber green mottle mosaic virus* (CGMMV) [4,14].

Melons are one of the most important horticultural crops in the world. International trade has increased the exchange of seeds and plant materials around the world and greatly promoted the spread of viruses on the global scale [16]. CGMMV, a highly destructive virus for melon crops, is one of the key seed-borne viruses in the ISTA certification protocol [17]. The main transmission routes of CGMMV are pollen and seed transmission [18] and mechanical transmission [6]; CGMMV infection seriously threatens the production of watermelon (*Citrullus lanatus*), melon (*Cucumis melo*), cucumber (*Cucumis sativus Linn*.), zucchini (*Cucurbita pepo L*.), and other crops. As a result, the yield of melons has decreased significantly [19,20], which has caused significant economic losses in some countries and regions [3,21,22].

The present study focused on the detection of CGMMV in plant seeds. The RT-qPCR reaction protocol was modified, and one-step pre-amplification reaction steps were established to accelerate nucleic acid cleavage and template enrichment in the samples to realize a more sensitivity of detection of the virus at low concentration in seeds. Simulated virus-contaminated seed powder samples were used to evaluate the sensitivity of detection of the improved one-step pre-amplification RT-qPCR assays in comparison with the universal RT-qPCR assays [14,24], universal RT-qPCR assays for double-quenchen probes [4] and DAS–ELISA assays [24,25].

## Results

### Homology analysis of target sequences

**Fig. 1:**
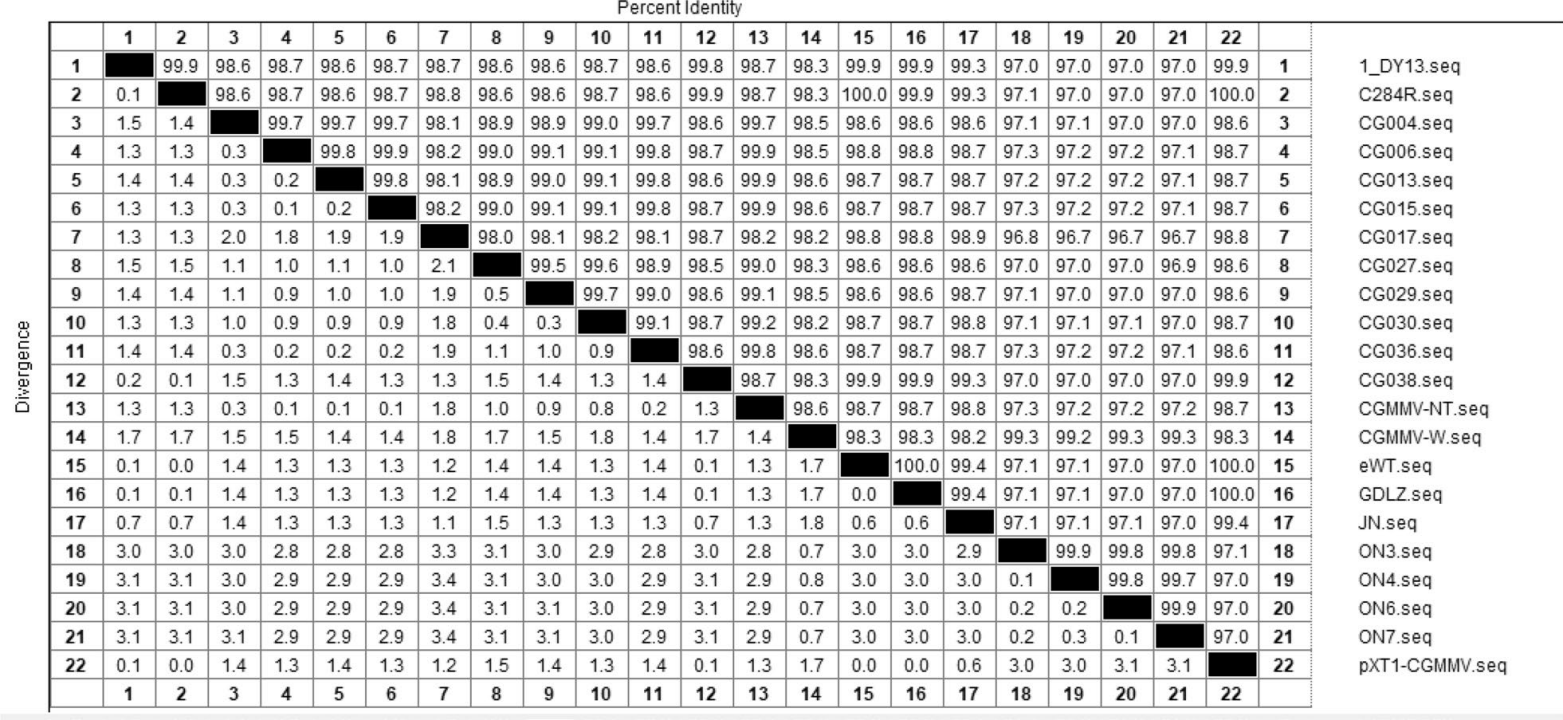
Comparative homology analysis of target gene sequences of 22 *Cucumber green mottle mosaic virus* (CGMMV) isolates using DNAStar software. As seen in the figure, the homology between 22 CGMMV isolates and DY13 isolate ranged from 97% to 99.9%

To design primers and probes (Table 1) for detection, highly conserved genomic regions were selected. The identity of 22 CGMMV isolates were shown in Additional file 1: Table S1. An alignment of the genome sequences of CGMMV isolate DY13 and 21 other CGMMV isolates showed that a region within the gene encoding the 129KD protein was completely conserved in all isolates. The homology results were shown in Fig. 1. DY13 showed 97.0–99.9% homology with 21 other CGMMV isolates, with the highest and lowest homology with the C284R (99.9%) and lowest pXT1 (97.0%) strains, respectively. The genomic target sequence was used to design primers and probes for the RT-qPCR detection of CGMMV.

**Table 1:** Primers and probes used for the detection of CGMMV in seeds

Primer	Sequence (5′-3′)	Position	Size of the PCR product (bp)	Reference
Fw1	ATCCGGAGTTTTCGATTA	257–274	96	This work
Rev1	GCATCATCATATATTCCAATTC	331–352		
Probe1	FAM-TTACCGCCACCAAGAACTCTGT-BHQ1	278–299		
Fw2	GCATAGTGCTTTCCCGTTCAC	6284–6304	101	[14, 24]
Rev2	TGCAGAATTACTGCCCATAGAAAC	6361–6384		
Probe2	FAM-CGGTTTGCTCATTGGTTTGCGGA-BHQ1	6315–6337		
Fw3	TTGGGCGTTGTGGTTTCTG	5209–5227	90	[4]
Rev3	AGAATGCATCCTTGTGTCAACC	5277–5298		
Probe3	FAM-AATCCTGTAGGGGTGGTGCTACTGT-DQ1	5245–5270		

*CGMMV* *Cucumber green mottle mosaic virus*

### Specificity of RT-qPCR assay

**Fig. 2:**
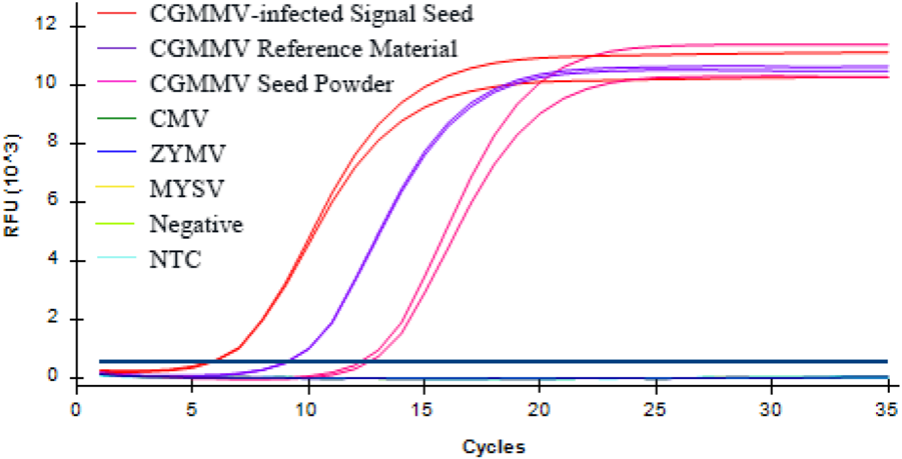
*Cucumber green mottle mosaic virus* (CGMMV) was detected by improved one-step pre-amplification RT-qPCR. CGMMV-infected single seed sample; CGMMV reference material; simulated CGMMV-contaminated seed powder base material; *cucumber mosaic virus* (CMV); *zucchini yellow mosaic virus* (ZYMV); *melon yellow spot virus* (MYSV). Tests were performed using total RNA extracted from leaves and seeds infected with one of the four viruses studied. For each assay, RNA extracted from uninfected seeds was used as negative control, along with a non-template control (NTC). Amplification plots show the normalized fluorescence values (ΔRn) versus the amplification cycle number, and horizontal lines denote the threshold limit of the test. Samples were tested in duplicate. Amplification plots correspond to improved one-step pre-amplification RT-qPCR

In this study, the detection specificity of the improved one-step pre-amplification RT-qPCR method was evaluated. We tested the specifificity of one-step pre-amplification RT-qPCR assay (Fig. 2) and the universal RT-qPCR assay (Figure S2) for CGMMV, respectively. The specificity of both the improved one-step pre-amplification RT-qPCR assay and universal RT-qPCR assay were analyzed using CGMMV-infected single seed, simulated virus-contaminated seed powder base material (the percentage of seeds infected with CGMMV in the total weight of zucchini seeds was 1%), and CGMMV positive control samples. Positive results were found on CGMMV, whereas no cross reaction of the other with 3 other viruses, including *Cucumber mosaic virus* (CMV), *Zucchini yellow mosaic virus* (ZYMV), and *Melon yellow spot virus* (MYSV), and healthy seed powder (Fig. 2, Additional file 1: Fig. S1).

In addition, the Ct value comparison between CGMMV-infected single zucchini seed powder sample and simulated virus-contaminated seed powder base material showed that the Ct value was higher for the latter.

### Sensitivity of RT-qPCR to detect simulated virus-contaminated seed powder

In this study, 10 groups of simulated virus-contaminated seed powder samples with different mass ratios were used as test materials, and four methods were used (Table 2). With the improved one-step pre-amplification RT-qPCR (Method A), the samples with the mass ratio 1:1000 produced a fluorescence amplification curve (Additional file 1: Fig. S2), that was, a single infected seed in 1000 seeds could be detected with this method. The linear standard curve equation was y = 51.501–20.277 lgX (R^2^ = 0.999), and the LOD reached 0.1%. Based on Chen et al. [14,24] universal RT-qPCR (Method B) and Torre et al. [4] universal RT-qPCR for double-quenchen probes (Method C), samples with a mass ratio of 1:900 could be detected as positive (Additional file 1: Fig. S3, S4), that was, a single infected seed in 900 seeds could be detected with universal RT-qPCR. The linear standard curve equation were y = 50.347–11.681 lgX (R^2^ = 0.928), y = 40.029–3.479 lgX (R^2^ = 0.998), respectively, and the LOD were 0.111%. However, the lowest mass ratio that was detected as positive by DAS–ELISA (Method D) was 1:500.

**Table 2:** CGMMV detection by four methods in simulated virus-contaminated seed powder samples with different mass ratios

Infected: healthy seed mass ratio	Mass percentage (%)^b^	Method A Ct Value ± *SD* ^c^	Method B Ct Value ± *SD*	Method C Ct Value ± *SD*	Method D
1:100	1	14.13 ± 0.04	27.58 ± 0.08	/^f^	+ ^d^
1:200	0.5	17.05 ± 0.00	29.45 ± 0.01	/	+
1:300	0.333	20.78 ± 0.02	32.78 ± 0.00	/	+
1:400	0.25	23.10 ± 0.04	34.02 ± 0.01	/	+
1:500	0.2	25.13 ± 0.01	35.41 ± 0.02	30.70 ± 0.25	+
1:600	0.167	25.72 ± 0.01	36.01 ± 0.12	33.20 ± 0.21	Undet
1:700	0.143	26.50 ± 0.06	36.57 ± 0.06	36.47 ± 0.31	Undet
1:800	0.125	27.40 ± 0.01	37.37 ± 0.52	36.74 ± 0.12	Undet
1:900	0.111	28.35 ± 0.23	38.64 ± 0.11	37.94 ± 0.40	Undet
1:1000	0.1	29.18 ± 0.98	Undet	Undet	Undet
Healthy	0	Undet^e^	Undet	Undet	Undet
Negative	0	Undet	Undet	Undet	Undet

*CGMMV* *Cucumber green mottle mosaic virus*

^a^The three methods: (A) one-step pre-amplification RT-qPCR; (B) universal RT-qPCR [14, 24]; (C) Universal RT-qPCR for double-quenchen probes [4]; (D) DAS–ELISA

^b^Mass percentage of infected seeds in total seeds, %

^c^Average Ct value of two replicates ± *SD*

^d^ + , positive

^e^Undet., no amplification detected

^f^Untested

### Uniformity evaluation of simulated low-concentration virus-contaminated seed powder samples

The simulated virus-contaminated seed powder samples with the mass ratios of 1:900 and 1:1000 were tested by the improved one-step pre-amplification RT-qPCR assay, the Ct values were determined, and the sample uniformity within and between vials were analyzed by one-way ANOVA. The uniformity test data were summarized in Additional file 1: Table S2 and Table S3, and the statistical analysis results of one-way ANOVA were listed in Table 3. At 95% confidence probability, the statistical *F* values of the two low-concentration virus-infected seed powder samples were 1.16 and 1.25, respectively, both of which were lower than *F*_0.05(19,20)_; thus, the non-uniformity of the sample was acceptable compared with the influence of other factors on the test results (Table 5). The results of analysis of variance (ANOVA) showed that the two groups of low-concentration virus-infected seed powder samples had good uniformity.

**Table 3:** Evaluation of uniformity in low-concentration CGMMV-infected seed powder samples

Infected: healthy seed mass ratio	Group	Sum of squares	Degrees of freedom	Mean square	*F* ratio	*F* Critical value	Confidence probability	Sample standard deviation
1: 900	Between groups	1.332860	19	0.070151	1.16	2.09	0.95	0.069
	Within group	1.212300	20	0.060615				
1: 1000	Between groups	1.510848	19	0.079518	1.25	2.09	0.95	0.089
	Within group	1.276850	20	0.063843				

*CGMMV* *Cucumber green mottle mosaic virus*

### Comparison of four methods for detection of low-concentration virus-infected seed powder samples

Uniformly prepared samples of low-concentration virus-infected seed powder (mass ratios of 1:900 and 1:1000) were tested using four methods, respectively (Table 4). The results suggested that the improved one-step pre-amplification RT-qPCR (Method A) detected all sub samples were CGMMV positive in 20 sub samples (*m* = 20) of low-concentration virus-infected seed powder with a mass ratio of 1:1000. However, Chen et al. [14, 24] universal RT-qPCR (Method B) could not detect CGMMV in low-concentration virus-infected seed powder with a mass ratio of 1:1000, and Torre et al. [4] universal RT-qPCR for double-quenchen probes (Method C) could detect two sub samples were CGMMV positive from 20 sub samples (*m* = 20), which might be the double-quenching probe could reduce the background fluorescence and improve the analytical sensitivity. DAS–ELISA (Method D) was not positive for the 20 subsamples in the two samples.

**Table 4:** CGMMV detection by four methods in low-concentration virus-infected seed

Infected: healthy seed mass ratio	Mass percentage (%) ^b^	Subsamples^c^ (m)	Percentage of positive results^d^ (positive detection rate, % ^e^)			
			Method A	Method B	Method C	Method D
1:900	0.111%	20	20 (100%)	20 (100%)	20 (100%)	0 (0%)
1:1000	0.1%	20	20 (100%)	0 (0%)	2 (10%)	0 (0%)
LOD (mass percentage, %)^f^			0.1	0.111	0.111	N.T. ^g^

*CGMMV* *Cucumber green mottle mosaic virus*, *LOD* limit of detection

^a^The four methods: (A) one-step pre-amplification RT-qPCR; (B) universal RT-qPCR [14, 24]; (C) universal RT-qPCR for double-quenchen probes [4]; (D) DAS–ELISA

^b^Mass percentage of infected seeds in total seeds, %

^c^number of subsamples, *m* = 20

^d^Number of positive results among 20 subsamples

^e^Percentage of the number of positive results detected in the total number of subsamples, %

^f^Under the 95% confidence probability, the positive number of 20 sub samples is detected

^g^N.T., not tested

### Actual sample testing

In this study, 33 samples of zucchini, cucumber, and pumpkin seeds (240 g each; equivalent to 2000–3000 seeds) were divided into 20 subsamples each (*m* = 20) and tested using the above-mentioned three methods (Table 5). Three batches of Zucchini seeds each out of 33 batches of seeds were detected as CGMMV-positive seeds by the improved pre-amplification RT-qPCR (Method A) and universal RT-qPCR methods (Method B) and were confirmed as CGMMV positive by further gene sequencing [24, 25]. The positive detection rate in 20 subsamples per sample of CGMMV-infected seeds by the improved one-step pre-amplification RT-qPCR (Method A) was > 95%. The positive detection rate in 20 subsamples per sample by universal RT-qPCR (Method B) was 10–55%. However, only two samples of CGMMV-infected seeds were detected as positive by DAS–ELISA (Method C), and one batch sample of low-concentration CGMMV-infected seeds could not be detected as positive.

**Table 5:** Detection of CGMMV in 33 batches of seeds using three detection methods

Samples	Number^b^ (batch)	Subsamples^c^/batch (m)	Number of positive results^d^ (Positive number/total number^e^)		
			Method A	Method B	Method C
Zucchini seeds	13	20	3(20/20;20/20;19/20)	3(20/20;11/20;2/20)	2(20/20;7/20)
Cucumber seeds	15	20	0	0	0
Pumpkin Seeds	5	20	0	0	0

*CGMMV Cucumber green mottle mosaic virus LOD* limit of detection

^a^The three methods: (A) pre-amplification RT-qPCR; (B) universal RT-qPCR [14, 24]; (C) DAS–ELISA

^b^Number of seed batches

^c^Each batch of seeds is divided into 20 subsamples, *m* = 20

^d^Number of positive results from 20 subsamples

^e^Ratio of the number of positive results to the number of subsamples

## Discussion

Detecting pathogens from seeds containing low concentrations of virus was key to preventing and controlling the spread of plant viruses carried by seeds [3]. ISTA requires that CGMMV in melon seeds (2000 seeds divided into 20 subsamples, with 100 seeds per subsample) be detected by DAS–ELISA [30]. According to the phytosanitary requirements for imported tomato and capsicum seeds issued by China [31], the seeds of tomato and capsicum exported to China should be tested for viruses using RT-PCR or RT-qPCR, and at least 3000 seeds for large quantities and at least 10% for small quantities of seeds should be used for the testing. Due to the different sizes of seeds, the weight of 2000 melon seeds were approximately 240 g.

In this study, an improved one-step pre-amplification RT-qPCR method for CGMMV detection was established, which could detect a single CGMMV-infected seed from 1000 seeds and had good sensitivity to detect low concentrations of virus in seeds. This method was more suitable for the detection of large quantities of seeds and seeds containing low concentrations of virus. The results suggested that using a pre-amplification stage in qPCR and not collecting fluorescence signals in the pre-amplification stage accelerated nucleic acid cleavage in samples and template enrichment, which was more conducive to the early observation of a positive fluorescence signal in the amplification stage of qPCR.

It was worth noting that the Ct value comparison between CGMMV-infected single zucchini seed powder sample and simulated virus-contaminated seed powder base material showed that the Ct value was higher for the latter. Therefore, this batch of seed substrates might be doped with healthy seeds or the CGMMV infection rate was low, and the virus-infected seeds were unevenly distributed in this group of seeds.

In addition, the specificity test results suggested high specificity of the designed probes in this study, which was consistent with the detection results established by Chen et al. [14, 24].

Compared with immunological detection techniques such as DAS-ELISA, RT-qPCR demonstrates higher sensitivity and specificity in detecting pathogens in plants [4, 32, 33]. Torre et al. [4] used RT-qPCR for double-quenchen probes and DAS–ELISA to detect CGMMV-infected cucumber seeds and showed that the detection sensitivity of RT-qPCR was 10 000 times higher than that of DAS–ELISA. In this study, the detection of seed samples with low concentrations of CGMMV demonstrated that the improved one-step pre-amplification RT-qPCR, the samples with the mass ratio 1:1000 produced a fluorescence amplification curve, that was, the LOD was 0.1%. Based on the Chen et al. [14, 24] universal RT-qPCR and Torre et al. [4] universal RT-qPCR for double-quenchen probes, samples with a mass ratio of 1:900 could be detected as CGMMV positive, that was, the LOD were 0.111%. However, the lowest mass ratio that was detected as positive by DAS–ELISA was 1:500. These results showed that the improved one-step pre-amplification RT-qPCR had significantly higher detection sensitivity than universal RT-qPCR and DAS–ELISA. The results suggested that the improved one-step pre-amplification RT-qPCR was more suitable for the detection of low-concentration CGMMV-infected seeds, had the advantage of higher sensitivity, and was suitable for virus detection in large quantities of seeds. The results supported that the improved pre-amplified RT-qPCR assay had higher sensitivity than universal RT-qPCR assay. In the present study, three batch CGMMV-infected seed samples from 33 batch seed samples were identified by improved one-step pre-amplification RT-qPCR. The positive detection rate of 20 subsamples of CGMMV-infected seeds was 95%, which suggested that the number of subsamples per batch seed samples could be reduced from 20 to 2–10, which could still ensure effective detection of seeds containing low concentrations of CGMMV. Generally, the virus carrying rate of virus infected seeds in seed batch was relatively low, which requiredd highly sensitive detection methods. The improved one-step pre-amplification RT-qPCR method had higher detection sensitivity, which was of great significance to prevent the spread of CGMMV-infected seeds to healthy production areas.

## Conclusions

In conclusion, the improved one-step pre-amplification RT-qPCR method designed and evaluated in this work have demonstrated their suitability as highly sensitive techniques for the evaluation of seed lots for CGMMV. Therefore, the implementation of these assays in cucurbit seed certification programmes could be seriously considered. This study provided a new idea for the detection of low concentration virus in seeds. Theoretically, this method could be applied to all low concentration virus detection to improve the detection sensitivity. It was of great significance for the detection of low concentration virus.

## Methods

### Plant material

CGMMV-infected zucchini seeds and healthy zucchini seeds were provided by Dalian Boli Biotechnology Co., Ltd (Dalian, China). The healthy zucchini seeds were determined to be negative for CGMMV by universal RT-qPCR and DAS-ELISA. Positive control samples for CGMMV, MYSV, and CMV were provided by Technology Center of Xiamen Customs District, (Xiamen, China). Positive control samples (C2300) for ZYMV was purchased from Agdia (Elkhart, IN, USA). In each test, total RNA from healthy seeds and positive control samples for MYSV, CMV and ZYMV were included as negative controls, and a blank control (double-distilled water) was also used. In total, 33 batches of zucchini seeds, cucumber seeds, pumpkin seeds, and other samples were purchased separately at the seed market in Liaoning, China.

### Primer and probe design

The whole genome sequence of the CGMMV isolate DY13 (Accession number: km873789.1) was retrieved from the National Center for Biotechnology and Information (NCBI) database and compared with the genome sequences of 21 other CGMMV isolates to identify highly conserved regions. Alignments were performed using the Clustal W method implemented in DNAStar MegAlign software. Primers and probes for the specific amplification of CGMMV were designed using Primer 5.0 software, and their specificity was evaluated with Basic Local Alignment Search Tool (BLAST) available at the NCBI website. The primers and probes were synthesized by TaKaRa Bio Inc. (Dalian, China).

### Simulated virus-contaminated seed powder preparation

Two methods were used for preparing the seed material for RNA extraction: (a) for the evaluation of individual infected seeds, each seed was frozen in liquid nitrogen and crushed in a mortar; (b) for large infected seed batches (100 seeds or more), an electric grinder (34BL99, Waring Blender Dynamics Corp., New Hartford, USA) was used. To prevent cross-contamination, both the mortar and grinder were cleaned with 0.2 M NaOH and rinsed with water between uses.

To prepare the virus-infected and healthy seed powder material, 150 zucchini seeds (approximately 20 g) infected with CGMMV and 20 000 healthy zucchini seeds (approximately 2400 g), respectively, were finely ground, and the ground material was passed through a 50-mesh sieve. The infected and healthy seed powder material were mixed in different mass ratios (1:100, 1:200, 1:300, 1:400, 1:500, 1:600, 1:700, 1:800, 1:900 and 1:1000) to obtain 10 batches of simulated virus-contaminated seed powder samples, which were used for RT-qPCR sensitivity detection. The percentage of seeds infected with CGMMV in the total weight of zucchini seeds was 1%, 0.5%, 0.333%, 0.25%, 0.20%, 0.167%, 0.143%, 0.125%, 0.111% and 0.1%, respectively. For the simulated virus-contaminated seed powder samples with the mass ratios 1:900 and 1:1000, 240 g (equivalent to 2000 zucchini seeds) of seed powder was prepared and aliquoted into 200 vials (1.2 g per vial). For the remaining mass ratios, 60 g seed powder was prepared and aliquoted into 50 vials (1.2 g per vial).

### RNA extraction

Total RNA extraction from large seed batches (150 mg of powdered seed material) was performed using the RNA Easy Fast Plant Tissue Kit (Code DP452, TianGen BiotechCO., LTD., Beijing, China) following the manufacturer’s instructions. Total RNA extraction from single seed powder sample (3 mg) was performed using the TaKaRa MiniBEST Viral RNA/DNA Extraction Kit Ver. 5.0 (Code 9766, TaKaRa Co., Ltd, Dalian, China)), following the manufacturer's instructions.

### RT-qPCR

One-step PrimeScript™ III RT-qPCR Mix (Code RR600A, TaKaRa Co., Ltd, Dalian, China) was used to perform RT-qPCR. For each reaction, 12.5 μL 2 × one-step PrimeScript™ III RT-qPCR mix, 0.5 μL primers (10 μM), 0.5 μL probe (10 μM), and 2 μL total RNA extract were used. The final reaction volume was 25 μL. The reactions were performed in a CFX96 Real-Time System (Bio-Rad, Hercules, CA, USA).

Chen et al. [14, 24] universal RT-qPCR, the following thermocycling conditions were used: A reverse transcription step of 5 min at 52 ℃, followed by PCR with a 10 s denaturation cycle at 95 ℃ and 40 amplification cycles of 5 s at 95 ℃ and 30 s at 60 ℃.

Torre et al. [4] universal RT-qPCR for double-quenchen probes, the following thermocycling conditions were used: A reverse transcription step of 5 min at 42 ℃, followed by PCR with a 3 min denaturation cycle at 95 ℃ and 40 amplification cycles of 3 s at 95 ℃ and 30 s at 60 ℃.

In this study, the universal RT-qPCR reaction procedure was modified, and the one-step pre-amplification reaction of qPCR was optimized. The optimized pre-amplification RT-qPCR thermocycling conditions were as follows: A reverse transcription step of 5 min at 52 ℃, followed by PCR with a 10 s denaturation cycle at 95 ℃, 15 amplification cycles of 5 s at 95 ℃ and 30 s at 60 ℃ during which fluorescence signals were not collected, and 40 amplification cycles of 5 s at 95 ℃ and 30 s at 60 ℃ during which fluorescence signals were collected.

### DAS-ELISA

Commercial ELISA Reagent Set for CGMMV (SRA45702, Agdia, Elkhart, IN, USA) Kit was used in this study. The DAS-ELISA procedure [24, 25] was the following: the prepared sample supernatant was added to the 96 well enzyme-linked plate coated with CGMMV antibody for DAS-ELISA detection. Each sample was subjected to double parallel test (*n* = 2). Healthy seeds are used as negative control, seeds infected with CGMMV were used as positive control, and the sample extraction buffer was used as blank control. Measure absorbance value with the iMark microplate absorbance reader at 405 nm at 30 min, 1 h and 2 h by iMark enzyme-linked detector (Bio-Rad, Hercules, CA, USA). If the absorbance value was 2 times higher than that obtained for the healthy control the sample was considerated positive. If the absorbance value was about 2 times but less than 2 times that of the healthy control group, the sample was considered suspected. If the absorbance value was 2 times lower than that obtained for the healthy control the sample was considerated negative.

### RT-qPCR sensitivity and specificity

The powder of single zucchini seeds infected with CGMMV; base material of simulated virus-contaminated seed powder (the percentage of seeds infected with CGMMV in the total weight of zucchini seeds was 1%); and positive control samples for CGMMV, MYSV, CMV, and ZYMV were used as test materials for to analyze the specificity of detection of RT-qPCR. Each sample was tested in duplicate (*n* = 2). The healthy seed powder material was used as the negative control, and water was used as the non-template control.

Simulated virus-contaminated seed powder samples with 10 different mass ratios were used to determine the limit of detection (LOD). Three methods were used for detection: (A) the improved pre-amplification RT-qPCR, (B) universal RT-qPCR [14, 24], (C) DAS–ELISA for CGMMV [24, 25] (SRA45702, Agdia, Elkhart, IN, USA). Each sample was tested in duplicate (*n* = 2).

### Uniformity test and detection comparison of simulated virus-contaminated low concentration seed powder samples

After the limit of detection (LOD) was determined by sensitivity test, simulated virus-contaminated seed powder samples with the mass ratio equivalent to the minimum detection limit and one higher mass ratio (20 random vials per sample; *m* = 20) were used to test the uniformity of the improved pre-amplification RT-qPCR method. Genomic RNA were extracted from each sample using the RNA Easy Fast Plant Tissue Kit (Code DP452, TianGen Biotech (Beijing) CO., LTD., China) according to manufacturers’ instructions. Each sample was tested in duplicate (n = 2).

Four methods were used to detect and compare the low concentration CGMMV seed powder samples: (A) the improved pre-amplified RT-qPCR assays, (B) Chen et al. [14, 24] universal RT-qPCR assays; (C) Torre et al. [4] universal RT-qPCR assays for double-quenchen probes; (D) DAS–ELISA for CGMMV [24, 25] (SRA45702, Agdia, Elkhart, IN, USA). Each batch of samples were randomly divided into 20 subsamples (*m* = 20), and each sub sample were repeated twice (*n* = 2).

### Statistical analysis

One-way ANOVA (*F*-test) was used to statistically analyze the uniformity of samples [26, 27]. The method of ISO13528:2005 was used to calculate the intra-unit (intra-vial) variance and inter-unit (inter-vial) variance [28], and finally calculate the *F* values. The critical value (*F*_0.05,(m-1),m(n-1)_) was obtained from the *F* distribution table [29]. When *F* ≤ *F*_0.05,(m-1),m(n-1)_, there was no significant difference between the inter-unit variance and intra-unit variance, indicating that the sample was uniform; When *F* > *F*_0.05,(m-1),m(n-1)_ there was a significant difference between the inter-unit variance and intra-unit variance, indicating that the sample was uneven [29].

### Actual sample testing

Zucchini seeds, cucumber seeds, and pumpkin seeds (33 samples) were purchased from the seed market in Liaoning, China. Each samples (240 g; equivalent to 2000 seeds) were crushed and fully mixed with a sterilized high-speed tissue crusher (34bl99, Waring Blender dynamics Corp., New Hartford, USA). Each samples were randomly divided into 20 sub samples (*m* = 20), each of which were tested in duplicate (*n* = 2). Total RNA (Code DP452, TianGen BiotechCO., LTD., Beijing, China) were extracted from each subsample. Three methods were used for detection: (A) the improved one-step pre-amplification RT-qPCR, (B) universal RT-qPCR [14, 24]; (C) DAS–ELISA for CGMMV [24, 25] (SRA45702, Agdia, Elkhart, IN, USA). The positive seed batches were further sequenced, and the gene sequencing were entrusted to TaKaRa Bio Inc. (Dalian, China).

## Supplementary Information

**Additional file 1:** **Figure S1.** *Cucumber green mottle mosaic virus* (CGMMV) was detected by universal RT-qPCR (Method B). **Figure S2.** Sensitivity assay of one-step pre-amplification RT-qPCR. Amplification plots show the testing of a serial dilution of infected zucchini seeds for the *Cucumber green mottle mosaic virus* (CGMMV). CGMMV-infected seed powder and healthy seed powder (passed through a 50-mesh sieve) were uniformly mixed according to mass ratios 1:100, 1:200, 1:300, 1:400, 1:500, 1:600, 1:700, 1:800, 1:900 and 1:1000. For each assay, RNA extracted from uninfected seeds was used as negative control, along with a non-template control (NTC). Plots show the normalized fluorescence values (ΔRn) versus the amplification cycle number and horizontal lines denote the threshold limit of the test. Sensitivity was estimated as the lowest concentration that produced an amplification signal in all replicates. **Figure S3.** Sensitivity assay of universal RT-qPCR (Method B). Amplification plots show the testing of a serial dilution of infected zucchini seeds for the *Cucumber green mottle mosaic virus* (CGMMV). **Figure S4.** Sensitivity assay of universal RT-qPCR for double-quenchen probes [4] (Method C). Amplification plots show the testing of a serial dilution of infected zucchini seeds for the *Cucumber green mottle mosaic virus* (CGMMV). **Table S1.** The identity of 22 CGMMV isolates. **Table S2.** Uniformity test in low-concentration CGMMV-infected seed powder samples (infected: healthy seed mass ratio = 1:900). **Table S3.** Uniformity test in low-concentration CGMMV-infected seed powder samples (infected: healthy seed mass ratio = 1:1000).

## Data Availability

All data generated or analysed during this study are included in this published article (and its Additional files).
