# Supplementary material for: Detection of Cucumber green mottle mosaic virus in low-concentration virus-infected seeds by improved one-step pre-amplification RT-qPCR
Source: Plant Methods. 2022 May 26;18:70. doi: 10.1186/s13007-022-00901-2 (PMC9134592; doi:10.1186/s13007-022-00901-2)

Supplementary materials

| Number | Isolates | Genbank | Source/Country | Collection date/year |
| --- | --- | --- | --- | --- |
| 1 | GDLZ | MK933286.1 | China | 2018 |
| 2 | NT | MH427279.1 | Australia | 2014 |
| 3 | CG038 | MH271443.1 | China | 2017 |
| 4 | CG036 | MH271441.1 | Canada | 2016 |
| 5 | CG030 | MH271435.1 | Greece | 2000 |
| 6 | CG029 | MH271434.1 | Greece | 2000 |
| 7 | CG027 | MH271432.1 | France | 2014 |
| 8 | CG017 | MH271422.1 | Thailand | 2016 |
| 9 | CG015 | MH271421.1 | Netherlands | 2016 |
| 10 | CG013 | MH271419.1 | Netherlands | 2016 |
| 11 | CG006 | MH271412.1 | Israel | 2015 |
| 12 | CG004 | MH271410.1 | USA | 2014 |
| 13 | ON7 | MF510469.1 | Canada | 2009 |
| 14 | ON6 | MF510468.1 | Canada | 2009 |
| 15 | ON4 | MF510466.1 | Canada | 2009 |
| 16 | ON3 | MF510465.1 | Canada | 2009 |
| 17 | pXT1 | KY753929.1 | China | 2011 |
| 18 | eWT | KY753928.1 | China | 2011 |
| 19 | C284R | KY753927.1 | China | 2011 |
| 20 | JN | KR232571.1 | China | 2015 |
| 21 | W | V01551.1 | Unknown | 2015 |
| 22 | DY13 | KM873789.1 | China | 2013 |

Table S1 The identity of 22 CGMMV isolates

Table S2 Uniformity test in low-concentration CGMMV-infected seed powder samples (infected: healthy seed mass ratio = 1:900)

| Sample number | Subsample 1 | Subsample 2 | Average value | Intra group bias 1 | Intra group bias 2 | Inter group bias | Intra group range |
| --- | --- | --- | --- | --- | --- | --- | --- |
|  | x_1j_ | x_2j_ | $\bar{x}_{j}$ |  |  |  | $\left(x_{1j}-x_{2j}\right)^{2}$ |
| 1 | 28.12 | 27.87 | 27.995 | 0.015625 | 0.015625 | 0.267912 | 0.062500 |
| 2 | 28.21 | 27.93 | 28.070 | 0.019600 | 0.019600 | 0.169362 | 0.078400 |
| 3 | 28.05 | 28.26 | 28.155 | 0.011025 | 0.011025 | 0.084872 | 0.044100 |
| 4 | 28.82 | 28.35 | 28.585 | 0.055225 | 0.055225 | 0.100352 | 0.220900 |
| 5 | 28.73 | 27.96 | 28.345 | 0.148225 | 0.148225 | 0.000512 | 0.592900 |
| 6 | 28.13 | 28.22 | 28.175 | 0.002025 | 0.002025 | 0.069192 | 0.008100 |
| 7 | 28.24 | 28.36 | 28.300 | 0.003600 | 0.003600 | 0.007442 | 0.014400 |
| 8 | 28.37 | 28.43 | 28.400 | 0.000900 | 0.000900 | 0.003042 | 0.003600 |
| 9 | 28.23 | 28.79 | 28.510 | 0.078400 | 0.078400 | 0.044402 | 0.313600 |
| 10 | 28.12 | 28.35 | 28.235 | 0.013225 | 0.013225 | 0.031752 | 0.052900 |
| 11 | 28.27 | 28.45 | 28.360 | 0.008100 | 0.008100 | 0.000002 | 0.032400 |
| 12 | 28.36 | 28.12 | 28.240 | 0.014400 | 0.014400 | 0.029282 | 0.057600 |
| 13 | 28.71 | 28.35 | 28.530 | 0.032400 | 0.032400 | 0.057122 | 0.129600 |
| 14 | 28.16 | 28.32 | 28.240 | 0.006400 | 0.006400 | 0.029282 | 0.025600 |
| 15 | 28.15 | 28.61 | 28.380 | 0.052900 | 0.052900 | 0.000722 | 0.211600 |
| 16 | 28.42 | 28.62 | 28.520 | 0.010000 | 0.010000 | 0.050562 | 0.040000 |
| 17 | 28.69 | 28.19 | 28.440 | 0.062500 | 0.062500 | 0.012482 | 0.250000 |
| 18 | 28.27 | 28.71 | 28.490 | 0.048400 | 0.048400 | 0.033282 | 0.193600 |
| 19 | 28.81 | 28.69 | 28.750 | 0.003600 | 0.003600 | 0.302642 | 0.014400 |
| 20 | 28.64 | 28.36 | 28.500 | 0.019600 | 0.019600 | 0.038642 | 0.078400 |

Abbreviations: CGMMV, *Cucumber green mottle mosaic virus*

Table S3 Uniformity test in low-concentration CGMMV-infected seed powder samples (infected: healthy seed mass ratio = 1:1000)

| Sample number | Subsample 1 | Subsample 2 | Average value | Intra group bias 1 | Intra group bias 2 | Inter group bias | Intra group range |
| --- | --- | --- | --- | --- | --- | --- | --- |
|  | x_1j_ | x_2j_ | $\bar{x}_{j}$ |  |  |  | $\left(x_{1j}-x_{2j}\right)^{2}$ |
| 1 | 29.34 | 29.17 | 29.255 | 0.007225 | 0.007225 | 0.040755 | 0.028900 |
| 2 | 28.32 | 28.97 | 28.645 | 0.105625 | 0.105625 | 0.436645 | 0.422500 |
| 3 | 28.98 | 29.23 | 29.105 | 0.015625 | 0.015625 | 0.000105 | 0.062500 |
| 4 | 29.26 | 28.83 | 29.045 | 0.046225 | 0.046225 | 0.009045 | 0.184900 |
| 5 | 29.31 | 28.85 | 29.080 | 0.052900 | 0.052900 | 0.002080 | 0.211600 |
| 6 | 28.89 | 29.13 | 29.010 | 0.014400 | 0.014400 | 0.020910 | 0.057600 |
| 7 | 29.45 | 29.61 | 29.530 | 0.006400 | 0.006400 | 0.349030 | 0.025600 |
| 8 | 29.32 | 29.47 | 29.395 | 0.005625 | 0.005625 | 0.159895 | 0.022500 |
| 9 | 28.81 | 29.33 | 29.070 | 0.067600 | 0.067600 | 0.003570 | 0.270400 |
| 10 | 29.44 | 28.94 | 29.190 | 0.062500 | 0.062500 | 0.012090 | 0.250000 |
| 11 | 29.36 | 29.21 | 29.285 | 0.005625 | 0.005625 | 0.059685 | 0.022500 |
| 12 | 28.83 | 28.93 | 28.880 | 0.002500 | 0.002500 | 0.107880 | 0.010000 |
| 13 | 29.16 | 29.31 | 29.235 | 0.005625 | 0.005625 | 0.030135 | 0.022500 |
| 14 | 29.57 | 28.79 | 29.180 | 0.152100 | 0.152100 | 0.009180 | 0.608400 |
| 15 | 29.11 | 29.23 | 29.170 | 0.003600 | 0.003600 | 0.006670 | 0.014400 |
| 16 | 29.09 | 28.91 | 29.000 | 0.008100 | 0.008100 | 0.025200 | 0.032400 |
| 17 | 29.32 | 29.15 | 29.235 | 0.007225 | 0.007225 | 0.030135 | 0.028900 |
| 18 | 29.09 | 28.83 | 28.960 | 0.016900 | 0.016900 | 0.046360 | 0.067600 |
| 19 | 29.36 | 28.93 | 29.145 | 0.046225 | 0.046225 | 0.002145 | 0.184900 |
| 20 | 28.91 | 28.75 | 28.830 | 0.006400 | 0.006400 | 0.159330 | 0.025600 |

Abbreviations: CGMMV, *Cucumber green mottle mosaic virus*

FIGURE S2


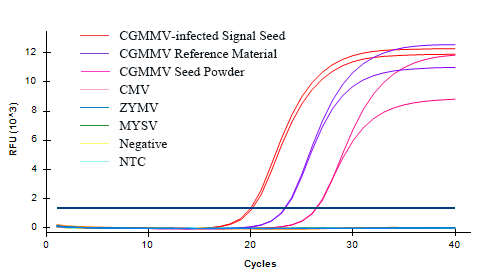


FIGURE S3


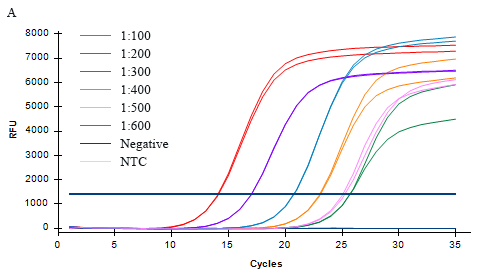

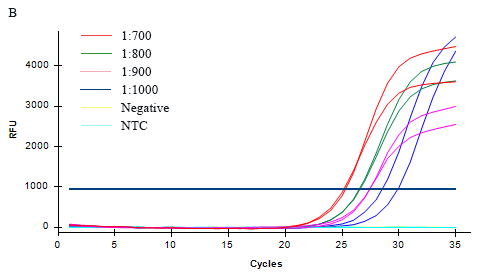


FIGURE S4


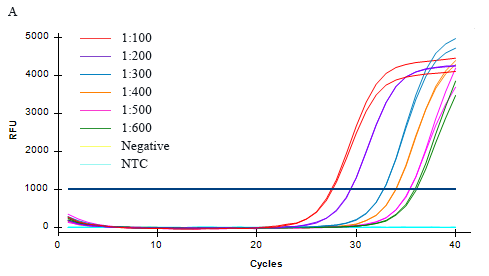

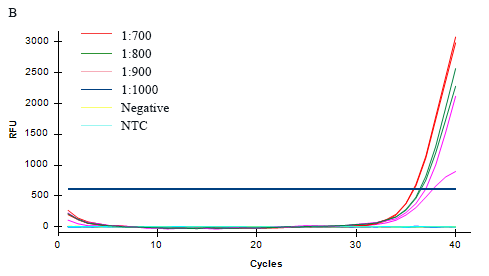


FIGURE S5


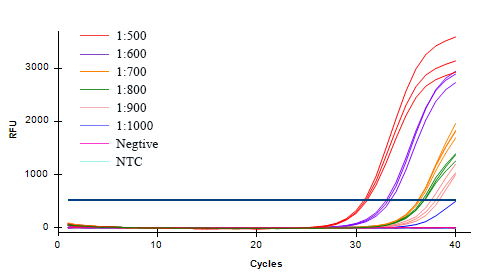

Supplement: Supplementary file 1 — Additional file 1: Figure S1. Cucumber green mottle mosaic virus (CGMMV) was detected by universal RT-qPCR (Method B). Figure S2. Sensitivity assay of one-step pre-amplification RT-qPCR. Amplification plots show the testing of a serial dilution of infected zucchini seeds for the Cucumber green mottle mosaic virus (CGMMV). CGMMV-infected seed powder and healthy seed powder (passed through a 50-mesh sieve) were uniformly mixed according to mass ratios 1:100, 1:200, 1:300, 1:400, 1:500, 1:600, 1:700, 1:800, 1:900 and 1:1000. For each assay, RNA extracted from uninfected seeds was used as negative control, along with a non-template control (NTC). Plots show the normalized fluorescence values (ΔRn) versus the amplification cycle number and horizontal lines denote the threshold limit of the test. Sensitivity was estimated as the lowest concentration that produced an amplification signal in all replicates. Figure S3. Sensitivity assay of universal RT-qPCR (Method B). Amplification plots show the testing of a serial dilution of infected zucchini seeds for the Cucumber green mottle mosaic virus (CGMMV). Figure S4. Sensitivity assay of universal RT-qPCR for double-quenchen probes [4] (Method C). Amplification plots show the testing of a serial dilution of infected zucchini seeds for the Cucumber green mottle mosaic virus (CGMMV). Table S1. The identity of 22 CGMMV isolates. Table S2. Uniformity test in low-concentration CGMMV-infected seed powder samples (infected: healthy seed mass ratio = 1:900). Table S3. Uniformity test in low-concentration CGMMV-infected seed powder samples (infected: healthy seed mass ratio = 1:1000). [file 13007_2022_901_MOESM1_ESM.docx]
